# Antibacterial and Chemical Characterization of Silica-Quercetin-PEG Hybrid Materials Synthesized by Sol–Gel Route

**DOI:** 10.3390/molecules27030979

**Published:** 2022-02-01

**Authors:** Ignazio Blanco, Alberta Latteri, Gianluca Cicala, Antonio D’Angelo, Veronica Viola, Vincenzo Arconati, Michelina Catauro

**Affiliations:** 1 Department of Civil Engineering and Architecture and INSTM UdR, University of Catania, V.le A. Doria 6, 95125 Catania, Italy; iblanco@unict.it (I.B.); alberta.latteri@unict.it (A.L.); gcicala@unict.it (G.C.); 2 Department of Engineering, University of Campania “Luigi Vanvitelli”, Via Roma 29, 81031 Aversa, Italy; antonio.dangelo@unicampania.it (A.D.); veronica.viola@unicampania.it (V.V.); vincenzo.arconati@gmail.com (V.A.); 3 Department of Environmental, Biological and Pharmaceutical Sciences and Technologies, University of Campania “Luigi Vanvitelli”, Via Vivaldi 43, 81100 Caserta, Italy

**Keywords:** sol–gel method, hybrids materials, anti-bacterial properties, FT-IR, SEM

## Abstract

This paper aims to synthesize, via the sol–gel method, a biomaterial usable in the medical field. Here, the silica-PEG-quercetin system was evaluated in relation to the different concentrations of PEG (0, 6, 12, 24, 50 wt%) and quercetin (0, 5, 10, 15 wt%), respectively. In addition, Fourier Transform-Infrared spectroscopy (FT-IR), Scanning Electron Microscopy (SEM), and Kirby–Bauer analyses were performed. FT-IR was used to evaluate the hybrid formation and the influence of both PEG and Quercetin in the hybrid synthesized materials, SEM was used to evaluate the morphological properties, while the Kirby–Bauer test was used to understand the ability of the materials to inhibit the growth of the assayed bacterial strains *(Escherichia coli*, *Pseudomonas aeruginosa*, *Enterococcus faecalis*, and *Staphylococcus aureus*).

## 1. Introduction

**Figure 1:**
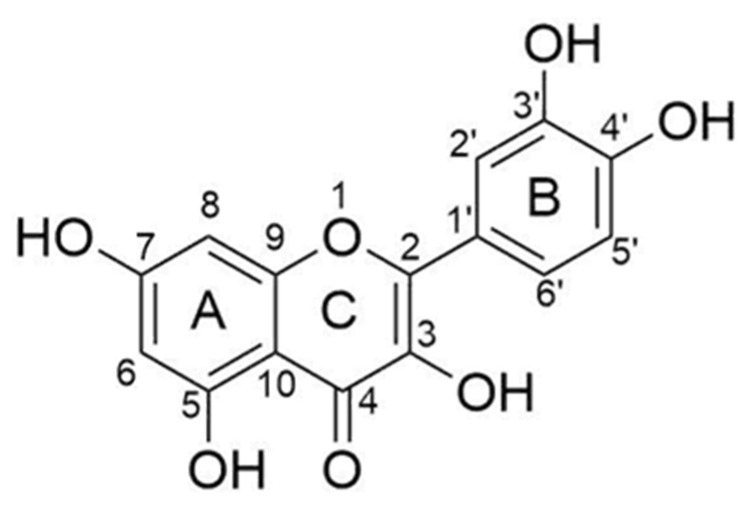
Chemical structure of quercetin.

Flavonoids are a varying group of natural substances, also called phytonutrients. They belong to a class of secondary metabolites having a polyphenolic structure, widely found in the plant kingdom. In particular, flavonoid compounds can be found in several parts of the plants, such as fruits, leaves, stems, bark roots, and flowers [1,2]. Flavonoids are divided into several groups depending on the modifications of the central C-ring. There are six different subclasses, such as flavones, flavonols, flavanones, flavanols (catechins), anthocyanidins, and isoflavones [1,3]. Among the flavonol subclass, quercetin is the main and the most thoroughly studied due to its anti-allergic, anti-bacterial, anti-oxidant, anti-viral, anti-inflammatory, and anti-carcinogenic properties [1]. The beneficial properties of this compound are due to the chemical structure, especially for the presence and location of the hydroxyl (–OH) substitutions and the catechol-type B-ring. In particular, the presence of an ortho-dihydroxy or catechol group in the B-ring, a 2,3-double bond, and a hydroxyl substitution at positions 3 and 5 make quercetin a strong anti-oxidant, able to chelate metals and scavenge oxygen free radicals (Figure 1) [4,5].

Regarding the antibacterial properties, it was observed that a minimum of 20 μg/mL of quercetin is sufficient for the inhibition of Staphylococcus aureus and *Pseudomonas aeruginosa*; a minimum of 300 μg/mL for the inhibition of *Proteus vulgaris*, and a minimum of 400 μg/mL can inhibit Escherichia coli. Concentrations higher than 50 μg/mL have bactericidal proprieties for Staphylococcus aureus and Pseudomonas aeruginosa; higher than 500 μg/mL for *Proteus vulgaris* and *Escherichia coli* [6]. All these proprieties make quercetin a suitable compound to be used in biomaterials. Biomaterials, which are substances engineered to interact with biological systems for medical purposes [7], can be synthesized via the sol–gel method. This method consists of a set of hydrolysis and polycondensation reactions of metal alkoxides that allows the transition of a sol–gel system from a colloidal solution (the ‘sol’) into a solid ‘gel’ phase [8]. During this process, a polymer network is formed and the incorporation of a variety of compounds, such as quercetin, is possible [9]. Furthermore, much research has demonstrated that the introduction of polyethylene glycol (PEG) used as an organic additive in the sol–gel methods can protect and change the speed of drug release incorporated into the matrix [9,10,11].

Other studies demonstrated that the entrapment of quercetin in silica sol–gel matrix is possible, and it allows the materials to acquire anti-oxidant properties. Preliminary studies were focused on the ability of the synthesized biomaterials (with a low percentage of quercetin) to induce the formation of the hydroxyapatite layer, investigating the bioactivity [12]. Starting from these results, this paper aims to synthesize, via sol–gel methods, a biomaterial usable in the medical field, observing how different concentrations ratio of PEG and quercetin can influence the material as well as its antibacterial activity. Here, the silica-PEG-quercetin system was evaluated in relation to the different concentrations of PEG (0, 6, 12, 24, 50 wt%) and quercetin (0, 5, 10, 15 wt%), respectively. In addition, Fourier transform-Infrared spectroscopy (FT-IR), scanning electron microscopy (SEM), and Kirby–Bauer analyses were performed. FT-IR was used to evaluate the hybrid formation and the influence of both PEG and Quercetin in the synthesized hybrid materials, SEM was used to evaluate the morphological properties, while the Kirby–Bauer test was used to understand the ability of the material to inhibit bacteria (*Escherichia coli, Pseudomonas aeruginosa, Enterococcus faecalis,* and *Staphylococcus aureus*).

## 2. Results and Discussion

### 2.1. Silica/PEG/Quercetin System

**Figure 2:**
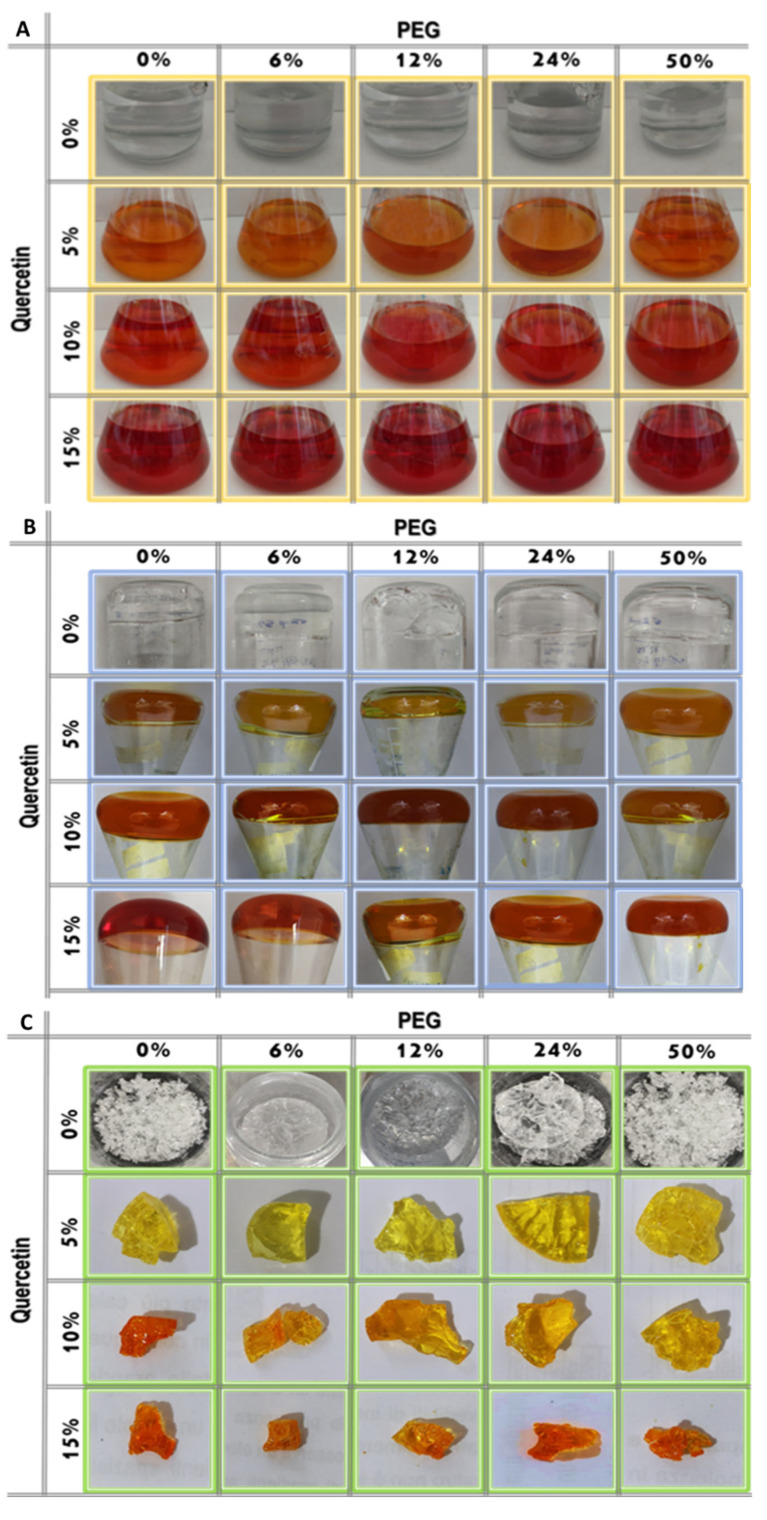
Sol–Gel step by step images of the synthesized samples: (**A**) “Sol” phase; (**B**) “Gel” phase and (**C**) the synthesized biomaterials.

Figure 2 reports the whole synthesis processes (from the “Sol” to the “Gel” and the “Drying” phases). Briefly, the solutions obtained in the absence of quercetin (SiO_2_/PEG (0, 6, 12, 24, and 50 wt%) were clear, transparent, and colorless, while the presence of quercetin amount (5, 10, and 15 wt%) is linked to an increase in the “yellow” color of the solutions, which appeared to be red at high quercetin amounts (Figure 2A). After the gelation occurrence, the samples with a lower amount of quercetin still remained yellow, and the ones with a higher amount were light orange (Figure 2B). After the heat treatment, all the synthesized glassy hybrid biomaterials, obtained in the presence of quercetin, appear to be with a yellow or a light orange color (Figure 2C). 

**Figure 3:**
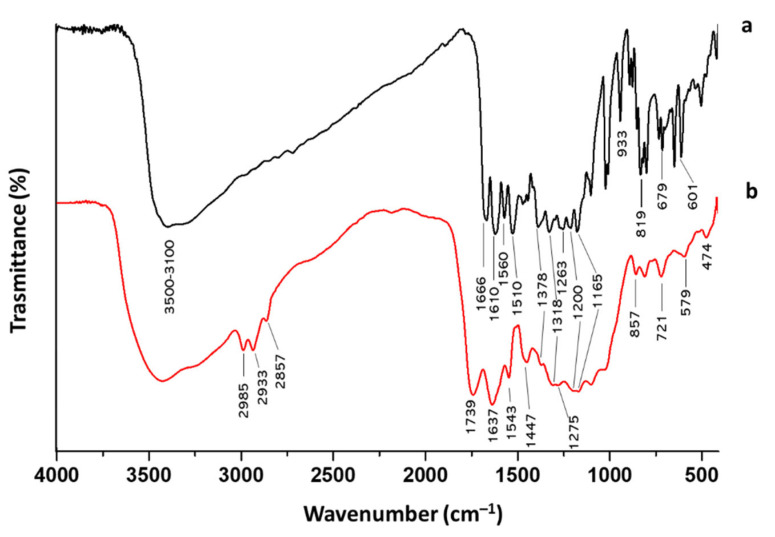
FT-IR spectra of quercetin (**a**) before and (**b**) after the Sol–Gel synthesis condition.

This different color is due to the oxidation of the quercetin structure during the synthesis, which can also be noticed by the differences that appear in the FT-IR spectra of quercetin before and after the synthesis condition (Figure 3). Indeed, the C=O band of the aryl ketonic group, assigned to 1666 cm^−1^, shifts to a higher wavenumber (1739 cm^−1^). This shift can be associated with the break of the double bond in the C-ring that reduces the electron delocalization of C=O. Moreover, this shift can also be attributed to the passage of the carbonyl group from a cyclohexanone to a cyclopentanone structure that causes an increase in its absorption frequency through a higher wavenumber value [13]. In addition, the passage toward cyclohexanone to cyclopentanone with higher bond tension causes the shift of aryl ether peak 1263 cm^−1^ to 1275 cm^−1^ [Silverstein]. The earlier macroscopic information about these obtained hybrids suggests that the Sol–Gel method was able to obtain a homogeneous biomaterial.

### 2.2. SEM Analysis of Silica/PEG/Quercetin

**Figure 4:**
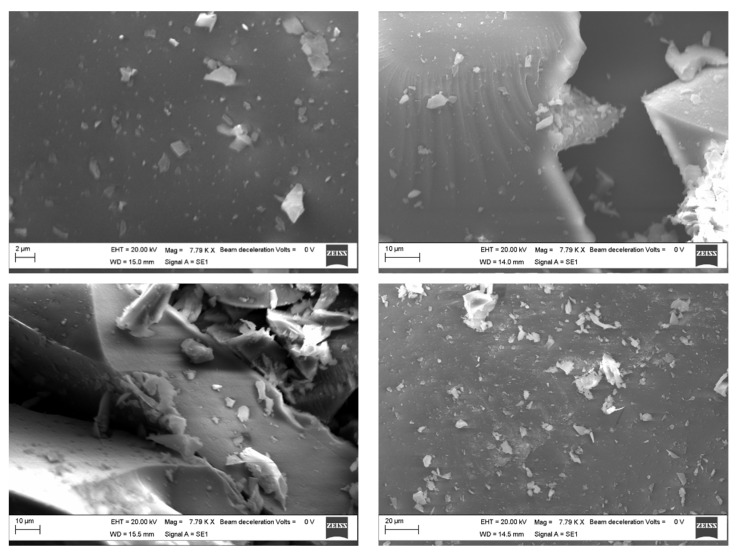
SEM images of the silica-PEG (12 wt%)-quercetin (10%) over 2 μm (**top left**) and 10 μm (**top right**) surface area and silica-PEG (24 wt%)-quercetin (10 wt%) over 10 μm (**bottom left**) and 20 μm (**bottom right**) surface area.

Scanning electron microscopy was carried out to have information about the morphology of the prepared samples. SEM images were collected over a selected area of 2–20 μm surface of the samples, not highlighting significant differences in the particle agglomeration as a function of the smaller (2 μm) or larger distance (20 μm), as we expected following the nanosilica functionalization [14]. Observing the micrographs of silica-PEG (12 wt%)-quercetin (10 wt%) and silica-PEG (24 wt%)-quercetin (10 wt%) (Figure 4), a good homogenization among the different materials of the composites can be observed, and the increase in size confirms the successful encapsulation of PEG and quercetin in the silica. In the surface morphology of the prepared samples, no appreciable difference among the different materials and compositions was observed. SEM investigation suggests that both PEG and quercetin embedded in the silica do not affect the morphology of the obtained hybrid composites. The organic and inorganic phases seem to be well homogenized, thus confirming the hybrid feature of the synthesized sol–gel materials

### 2.3. FT-IR Analysis of Silica/PEG/Quercetin

**Figure 5:**
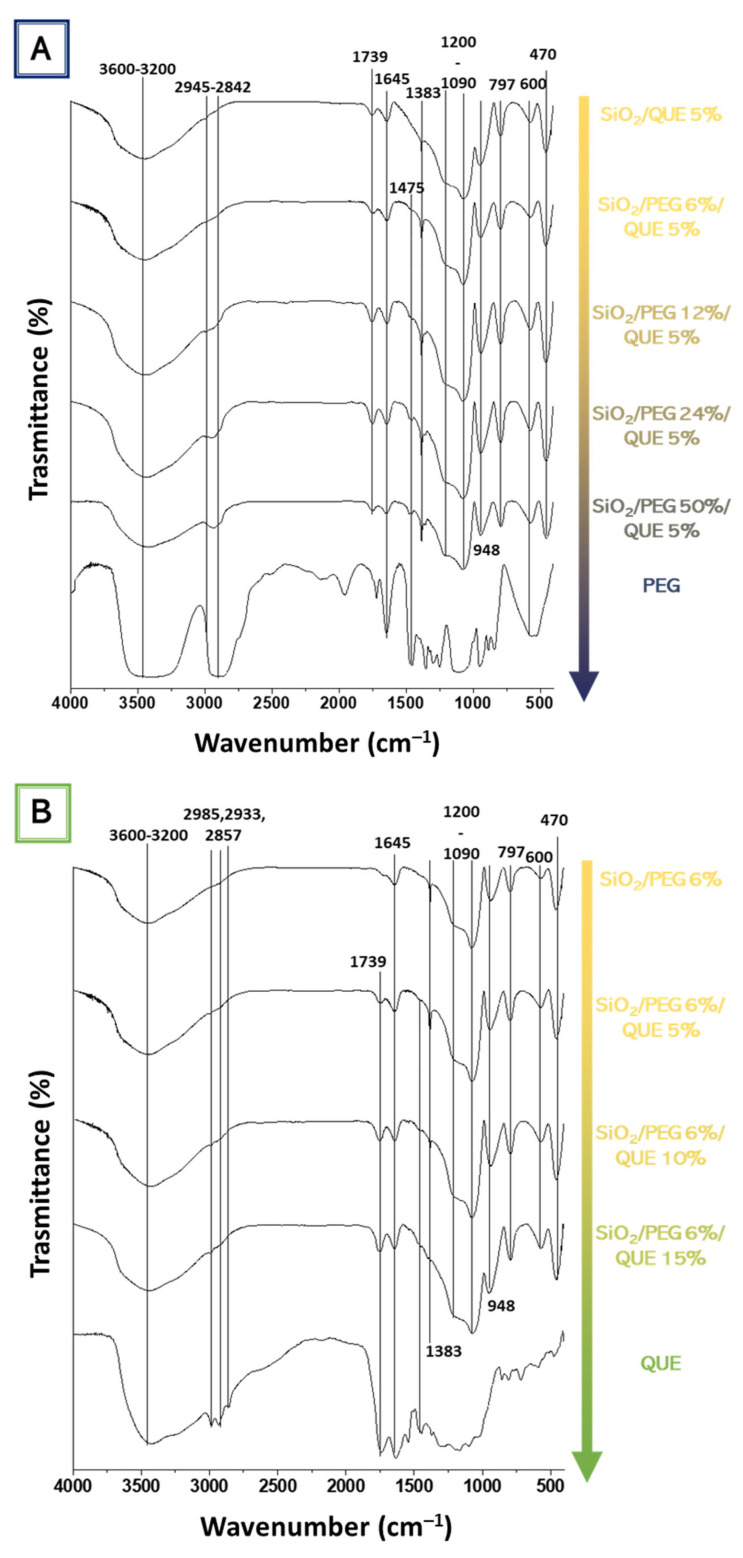
FT-IR spectra of the synthesized hybrid materials as a function of (**A**) PEG and (**B**) quercetin.

Figure 5A reports the FT-IR spectra of the hybrid systems based on SiO_2_/PEG (from 0 to 50 wt%)/Que (5 wt%) and PEG, while Figure 5B reports the FT-IR spectra of hybrid systems based on SiO_2_/PEG (6 wt%)/Que (from 0 to 15 wt%) and quercetin. The former figure pointed out the influence of the increasing PEG amount, whereas the latter underlines the quercetin influence in the synthesized biomaterials. As shown in Figure 5A, the bands at 3600-3200 cm^−1^ and 2880 cm^−1^, assigned to -OH and -CH_2_ stretching [11,13,15], became more intensive with the increase in the PEG content. Moreover, all the spectra also have the -OH bending vibration at 1650 cm^−1^, which peak is higher in the PEG spectrum. In Figure 5B, the influence of quercetin amount is followed by the growing peak at 1740 cm^−1^ due to the C=O vibration [13] of the C-ring. Moreover, in the hybrid’s material spectra (Figure 5B), this peak also possesses the same rounded shape as the one visible in the quercetin spectrum (Figure 5B). The spectra of the hybrid materials (both Figure 5A,B) reveal the Si-O-Si asymmetric stretching at 1090 cm^−1^ with a shoulder at 1200 cm^−1^ and the Si-O bending at 470 cm^−1^. The co-presence of quercetin, PEG, and silica peaks suggest the formation of the hybrids, in which both the organic and inorganic parts could interact with hydrogen bonds. More detailed data about the other peaks can be found elsewhere in our previous papers [13,14,15,16].

### 2.4. Antibacterial Properties of Silica/PEG/Quercetin

**Figure 6:**
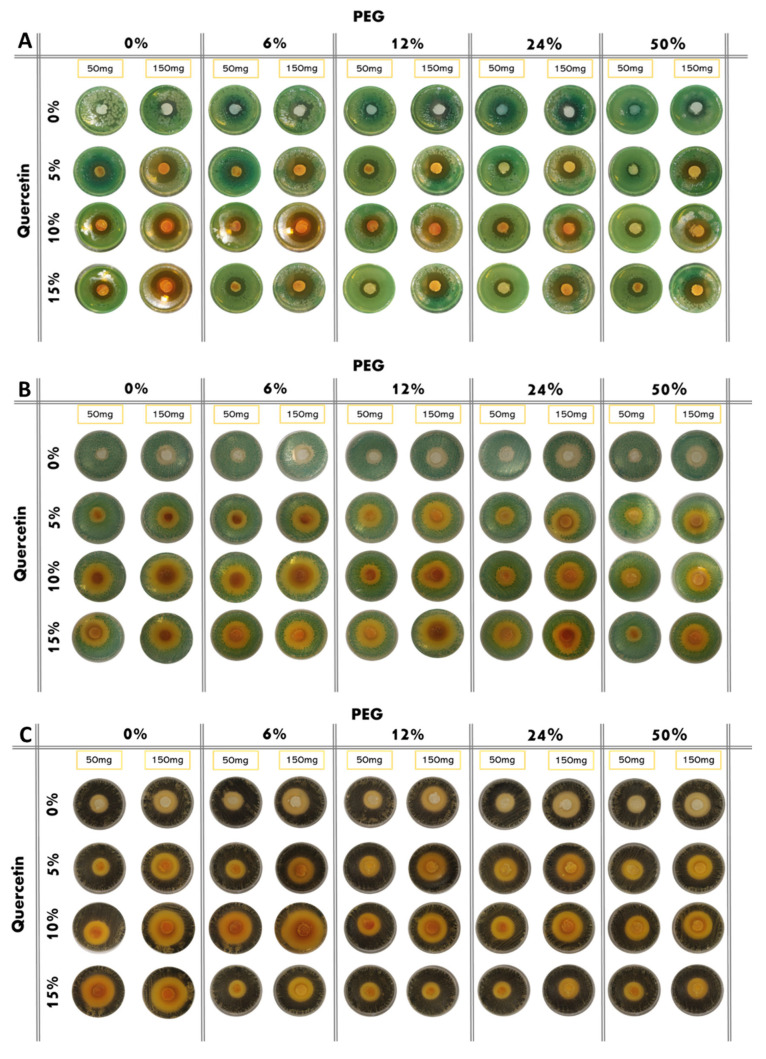

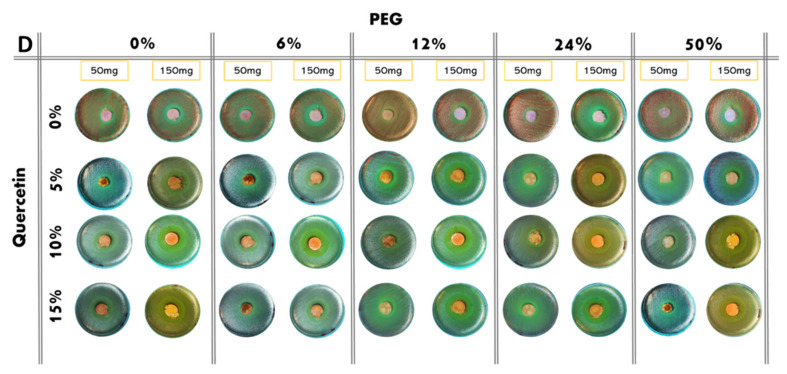
Inhibition images of samples assayed in the presence of: (**A**) *P. aeruginosa*; (**B**) *E. coli*; (**C**) *S. aureus*; (**D**) *E. faecalis*.

The results of the antibacterial test are shown in Figure 6. Based on the observation of the plates, all the bacteria, both Gram-positive and Gram-negative, are sensitive to the presence of the hybrid material. Indeed, it has been demonstrated that both PEG and quercetin possess anti-microbial activity [17,18]. Furthermore, in this paper, all the bacteria considered are dose-dependent: the inhibition halos are wider in the 150 mg samples than the 50 mg samples. A comparison of inhibition halos diameters produced by SiO_2_/PEG with different amounts of entrapped quercetin is shown in Figure 6A,B.

**Figure 7:**
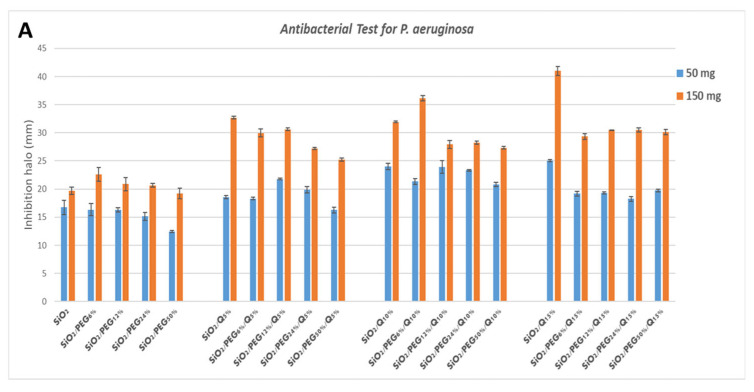

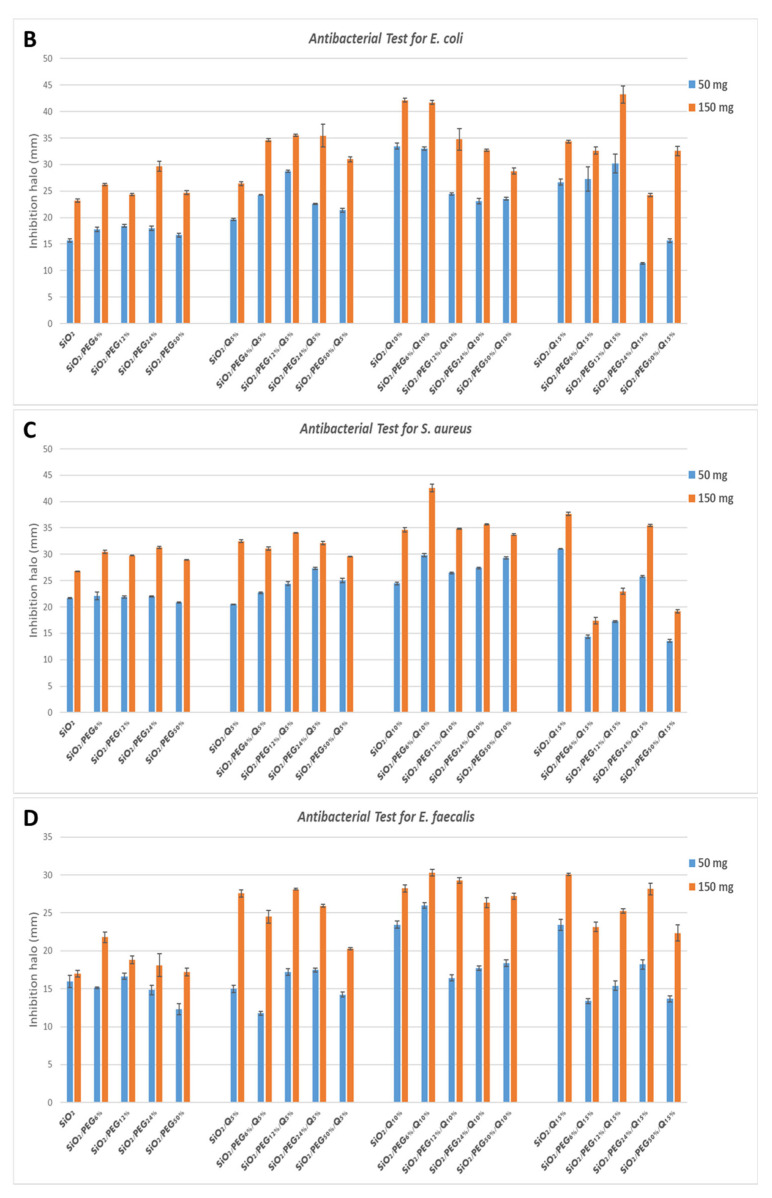
Inhibition halo histograms of samples assayed in the presence of: (**A**) *P. aeruginosa*; (**B**) *E. coli*; (**C**) *S. aureus;* (**D**) *E. faecalis*. Values are the means ± SD of measurements carried out on samples analyzed four times.

*P. aeruginosa* shows discrete sensitivity to the SiO_2_ that does not change much with the increment of the PEG in the system until the PEG reaches 50 wt% of the composition. In this case, the antibacterial activity seems to reduce. Conversely, as quercetin increases in the system, the inhibition halo also seems to increase. Further, in this case, the addition of PEG in the system causes a light reduction in the inhibition halos (Figure 7A).

*E. coli* shows an inhibition halo diameter increased when the quercetin is present in the system. The observation of the different samples shows that the best halos are obtained when the system is composed of SiO_2_/PEG 6 wt%/Que 10 wt% and SiO_2_/PEG 12 wt%/Que 15 wt% at 50 mg and 150 mg (Figure 7B), respectively. This figure also shows that hybrid material with a high amount of PEG causes a reduction in the antibacterial activity also when the amount of quercetin is high. The antibacterial test for *S. aureus* (Figure 7C) showed a constant inhibition halo when the system was based only on SiO_2_ and PEG, independently of the amount of SiO_2_ and PEG used. The hybrid material with the best anti-bacterial performance against *S. aureus* was the one with SiO_2_/PEG 6 wt%/Que 10 wt% compositions, while the samples with quercetin at 15 wt% and PEG in the range 6-50 wt% amounts obtained the worst performance. As the trends showed for the other tested bacterial strains, also the hybrid systems assayed in the presence of *E. faecalis* pointed out that the SiO_2_/PEG (6 wt%)/Que (10 and 15 wt%) had the highest efficiency. Indeed, 150 mg of both the systems plated in the middle of Petri plates had inhibition halos of 3.2 and 3.0 cm (Figure 7D), respectively.

## 3. Materials and Methods

### 3.1. Sol–Gel Synthesis

**Figure 8:**
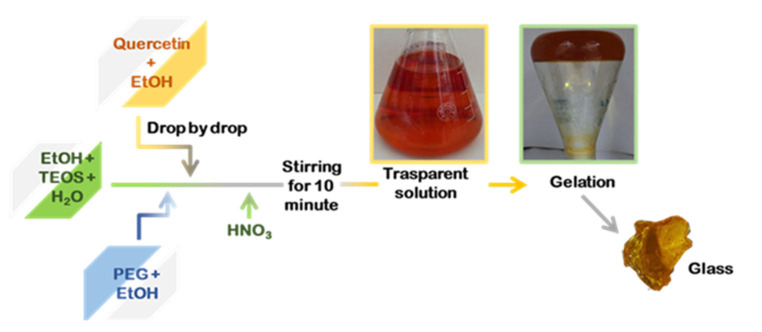
Flowchart procedure of the sol–gel synthesis.

Silica hybrid materials were synthesized by enriching them with different percentages of PEG (0, 6, 12, 24, 50 wt%) and quercetin (0, 5, 10 wt%) by the sol–gel methods. A flowchart of SiO_2_/PEG/quercetin synthesis is shown in Figure 8. Firstly, an inorganic silicate solution was obtained by adding tetraethyl orthosilicate (TEOS, Sigma-Aldrich, Darmstadt, Germany) to Ethanol (EtOH, Sigma-Aldrich) and the distillate water under continuous magnetic stirring. Secondly, a solution of quercetin followed by a solution of polyethylene glycol (PEG, MW = 400, Sigma-Aldrich), both solubilized in ethanol, were added to the system. Finally, nitric acid (<65%, Sigma-Aldrich) was added, and a clear and homogeneous solution was obtained after about 10 min of stirring. The different solutions prepared were put at room temperature until the gelation process was achieved. The gelled samples were dried at 50°C in the oven for 24 h, allowing the removal of the solvent residue.

### 3.2. Scanning Electron Microscopy

Morphology study of the prepared samples was carried out by SEM (ZEISS EVO MA 15, EVO-ZEISS, Cambridge, UK). The samples were gold-sputtered up to a thickness of 20 nm by means of an Emitech K-550 sputter coater (Ashford, Kent, UK). In order to perform the analysis, an accelerating voltage of 20.00 KV and a working distance of 15 mm was used. Scans were carried out at different magnifications ranging from 500× to 25,000×.

### 3.3. FT-IR Measurement

FT-IR analysis was made to investigate the chemical compositions of the materials and to identify the interactions among their components. The FT-IR transmittance spectra analysis, performed with Prestige21 Shimadzu system equipped with a DTGS KBr detector (deuterated triglycine sulfate with potassium bromide windows), were recorded in the range of 400–4000 cm^−1^ with a resolution of 2 cm^−1^ (60 scans). The analysis procedure used KBr disks in which 2 mg of sample were mixed with 200 mg of KBr. Prior to the analyses, the samples were dried in an oven at 105°C allowing the water removal. The elaboration of the FT-IR spectra was made by IR Solution and Origin 8 software.

### 3.4. Antibacterial Analysis

Preliminary information on antibacterial properties of glasses of Silica/PEG/Quercetin was obtained via anti-microbial susceptibility test. Both Gram-negative and Gram-positive bacteria were grown in the absence and presence of each sample. Specifically, *Escherichia coli* (ATCC 25922) and *Pseudomonas aeruginosa* (ATCC 27853) as Gram-negative bacteria and *Staphylococcus aureus* (ATCC 25923) and *Enterococcus faecalis* (ATTC 29212) as Gram-positive bacteria were used.

The whole procedure is divided into five steps: (i) media preparation, (ii) sample preparation, (iii) bacterial strains preparation, (iv) bacterial incubation, and (v) inhibition halo measurements.

TBX Medium (Tryptone Bile X-Gluc), Pseudomonas CN Agar, Baird–Parker Agar, and Slanetz–Bartley Agar Base, all purchased from Liofilchem (Roseto Degli Abruzzi, Italy), were prepared dissolving each nutrient in autoclaved water. Then all these bacterial media were sterilized at 120 °C for 15 min and slowly cooled. TBX and Slanetz–Bartley were poured directly into Petri dishes at 50 °C, while the Baird–Paker Agar and the Pseudomonas CN Agar were cooled up to 50 °C and the respective supplement nutrient (egg yolk containing potassium tellurite and pseudomonas supplement, respectively) were added before the solidification into the Petri dishes.

Sample disks (50 and 150 mg, respectively) were obtained by grounding and pressing the synthesized hybrid materials. Prior to the analysis, these disks were sterilized under UV light for 1 h.

The pellets of the bacterial strains were dissolved in distilled saline water (0.9% NaCl) and diluted, obtaining bacterial suspensions of 10^5^ CFU/mL, which were plated on the respective solid agar media. *E. coli* was plated on TBX Medium, *S. aureus* on Baird–Parker Agar, *P. aeruginosa* on Pseudomonas CN Agar, and *E. faecalis* on Slanetz–Bartley Agar Base.

Before the incubation time, the samples were placed in the middle of Petri dishes; after that, the bacteria were plated. *E. coli* was incubated at 44 °C for 24 h, *S. aureus* at 36°C for 24 h and *E. faecalis* and *P. aeruginosa* at 36 °C for 48 h.

Each sample was finely grounded and pressed, obtaining disks of 50 and 150 mg of weight, respectively. Sterilization then occurred with a UV light for about 1 h. The bacterial suspension of 10^5^ CFU/mL was obtained by diluting the strains in distilled saline water (0.9% NaCl). E. coli was plated on TBX Medium, (Tryptone Bile X-Gluc) (Liofilchem, Italy), S. aureus on Baird–Parker agar (Liofilchem, Italy), *P. aeruginosa* on Pseudomonas CN agar, and finally *E. faecalis* on Slanetz–Bartley agar base. All media, except for on Slanetz–Bartley agar base, were sterilized up to 120 °C for 15 min. After the sterilization process, Baird–Parker agar was cooled, leading it to the temperature of 50 °C, and an emulsion of egg yolk containing potassium tellurite was added. A CN pseudomonad supplement was also added to the pseudomonas CN agar when it was cooled up to 50/45 °C. The samples were placed in the middle of Petri dishes, and every dish was incubated. *E. coli* was incubated at 44 °C for 24 h while *S. aureus, E. faecalis,* and *P. aeruginosa* at 36 °C for 48 h. The diameters of the inhibition halos (IDs), after the incubation time, in relationship to Petri dish diameter (PPD) (6 cm) were calculated. For each sample, to determine the mean Standard Deviation, four measures of the diameter were taken.

## 4. Conclusions

The anti-microbial susceptibility test showed the antibacterial propriety of the samples. Independently from the composition, both Gram-positive (*S. aureus* and *E. faecalis*) and Gram-negative (*E. coli* and *P. aeruginosa*) bacteria are inhibited. These results pointed out that the presence of both quercetin and PEG, strongly affects the anti-bacterial properties of the synthesized materials. Indeed, there is no linear increment of inhibition halos by the increasing of the quercetin in the presence of PEG. This could be related to the entrapment of the quercetin into the porous systems that could slow down the release. Moreover, even though the FT-IR spectra underlined the formation of the hybrid materials by H-bonds and the SEM analysis confirmed a good homogenization of the materials enclosed in the prepared composites

Future studies will focus on the investigation of the kind of hybrid formed and also its ability to release the quercetin entrapped. This information could shed new light on the possibility of using these materials as active parts of biomaterials [19].

## Data Availability

No applicable.
